# Post-acute COVID-19 in three doses vaccinated autoimmune rheumatic diseases patients: frequency and pattern of this condition

**DOI:** 10.1186/s42358-023-00309-z

**Published:** 2023-06-08

**Authors:** Clovis Artur Silva, Leonard de Vinci Kanda Kupa, Ana Cristina Medeiros-Ribeiro, Sandra Gofinet Pasoto, Carla Gonçalves Schahin Saad, Emily Figueiredo Neves Yuki, Joaquim Ivo Vasques Dantas Landim, Victor Hugo Ferreira e Léda, Luisa Sacchi de Camargo Correia, Artur Fonseca Sartori, Carolina Campagnoli Machado Freire Martins, Carolina Torres Ribeiro, Filipe Waridel, Victor Adriano de Oliveira Martins, Samuel Katsuyuki Shinjo, Danieli Castro Oliveira Andrade, Percival Degrava Sampaio Barros, Eduardo Ferreira Borba Neto, Nadia Emi Aikawa, Eloisa Bonfa

**Affiliations:** 1 Rheumatology Division, Hospital das Clinicas HCFMUSP, Faculdade de Medicina, Universidade de Sao Paulo, Av. Dr. Arnaldo, 455, sala 3190 - Cerqueira César, São Paulo, SP 01246-903 Brazil; 2 Pediatric Rheumatology Unit, Instituto da Criança e do Adolescente, Hospital das Clinicas HCFMUSP, Faculdade de Medicina, Universidade de Sao Paulo, São Paulo, Brazil

**Keywords:** Autoimmune diseases, COVID-19, Long COVID-19, Post-COVID-19 syndrome

## Abstract

**Background:**

Data on post-acute COVID-19 in autoimmune rheumatic diseases (ARD) are scarce, focusing on a single disease, with variable definitions of this condition and time of vaccination. The aim of this study was to evaluate the frequency and pattern of post-acute COVID-19 in vaccinated patients with ARD using established diagnosis criteria.

**Methods:**

Retrospective evaluation of a prospective cohort of 108 ARD patients and 32 non-ARD controls, diagnosed with SARS-CoV-2 infection (RT-PCR/antigen test) after the third dose of the CoronaVac vaccine. Post-acute COVID-19 (≥ 4 weeks and > 12 weeks of SARS-CoV-2 symptoms) were registered according to the established international criteria.

**Results:**

ARD patients and non-ARD controls, balanced for age and sex, had high and comparable frequencies of ≥ 4 weeks post-acute COVID-19 (58.3% vs. 53.1%, *p* = 0.6854) and > 12 weeks post-acute COVID-19 (39.8% vs. 46.9%, *p* = 0.5419). Regarding ≥ 4 weeks post-acute COVID-19, frequencies of ≥ 3 symptoms were similar in ARD and non-ARD controls (54% vs. 41.2%, *p* = 0.7886), and this was also similar in > 12 weeks post-acute COVID-19 (68.3% vs. 88.2%, *p* = 0.1322). Further analysis of the risk factors for ≥ 4 weeks post-acute COVID-19 in ARD patients revealed that age, sex, clinical severity of COVID-19, reinfection, and autoimmune diseases were not associated with this condition (*p* > 0.05). The clinical manifestations of post-acute COVID-19 were similar in both groups (*p* > 0.05), with fatigue and memory loss being the most frequent manifestations.

**Conclusion:**

We provide novel data demonstrating that immune/inflammatory ARD disturbances after third dose vaccination do not seem to be a major determinant of post-acute COVID-19 since its pattern is very similar to that of the general population. *Clinical Trials* platform (NCT04754698).

**Supplementary Information:**

The online version contains supplementary material available at 10.1186/s42358-023-00309-z.

## Background

In October 2021, the World Health Organization (WHO) recognized a new clinical condition related to coronavirus disease 2019 (COVID-19) named post-COVID-19 syndrome [1]. Nalbandian et al. [2] defined post-acute COVID-19 as persistent symptoms and/or delayed or long-term complications of SARS-CoV-2 infection beyond 4 weeks from the onset of symptomatology.

This condition can further be divided into two categories: (1) subacute or ongoing symptomatic COVID-19, which includes symptoms and abnormalities present from 4 to 12 weeks beyond acute COVID-19 and (2) chronic or post-covid syndrome, which includes symptoms and abnormalities persisting beyond 12 weeks of the onset of acute COVID-19, not attributable to alternative diagnoses [2]. Both conditions are characterized by multisystemic involvement, with symptoms that occur during or after COVID-19 and persist for a prolonged time. The most common symptoms are chronic fatigue with post exertional features, neurocognitive dysfunction including memory and concentration deficit, and shortness of breath [2–5].

Post-acute COVID-19 was reported in 8–17% of the general population in a study that included more than one million individuals from England’s health system. The symptoms lasted > 12 weeks and were debilitating in 1.2–4.8% of cases. The risk factors identified were older age, female sex, Caucasian race, mental health disorder before the pandemic, obesity, and asthma, and no association was observed with the severity of the SARS-CoV-2 infection [3]. Contrasting with these data, a more significant number was reported *in the “(REACT)-2 Study” (Real-time Assessment of Community Transmission)* with approximately six hundred thousand participants, in which a frequency of 37.7% of post-acute COVID-19 was observed persisting for ≥ 12 weeks. Three or more manifestations were registered in 17.5% of the respondents [4].

Patients with autoimmune rheumatic diseases (ARD) have higher rates of SARS-CoV-2 infection and an increased mortality rate than the general population [6]. Data on post-acute COVID-19 in ARD patients are scarce [7–9], with ARD defined by the International Code of Diseases database, mostly focused on a single condition and variable definitions for this condition. These factors, associated with the heterogeneous time of vaccination in previous reports may have hampered the characterization of post-acute COVID-19 in ARD patients.

Therefore, the aim of this study was to characterize the post-acute COVID-19 in a well-established ARD patients sample and non-ARD controls concomitantly immunized with CoronaVac vaccine.

## Methods

### Study design

The present retrospective analysis was part of a prospective cohort that evaluated the immunogenicity and safety of the CoronaVac vaccine after the third dose [10]. The original study included 1048 ARD patients and 428 non-ARD controls. The control group included health care workers from Hospital das Clínicas da Faculdade de Medicina da Universidade de São Paulo. They were vaccinated along with the patients in February and March 2021 (first and second dose, respectively) and were followed concomitantly with the patients for additional doses, for SARS-CoV-2 infection from March 2021 to February 2022, and for post-acute COVID-19. The participants answered a structured questionnaire on SARS-CoV-2 infection and post-acute COVID-19 via telephone calls. The study was conducted in our single tertiary center, and the period of assessment was from September 18th, 2021 (date of the third vaccine dose) to February 28th, 2022. The inclusion criteria were patients with current age ≥ 18 years old and diagnosed with ARD according to the international classification criteria for each disease, as previously reported [10], and have received three doses of CoronaVac vaccination. Exclusion criteria were prior anaphylactic events to vaccines, immunisation with live virus in the last 4 weeks or inactivated vaccine in the last 2 weeks prior to vaccination, Guillain-Barré syndrome, decompensated heart failure demyelinating disease, COVID-19-related symptoms, acute febrile illness, or hospitalisation at vaccination day. Further exclusion criteria for this study included participants who did not answer properly the questionnaire on SARS-CoV-2 infection and post-acute COVID-19, or no confirmation of SARS-CoV-2 infection by RT-PCR or antigen testing. The project was approved by the National Local Ethics Committee (CAAE: 42,566,621.0.0000.0068) and was registered on *the Clinical Trials* platform (NCT04754698).

### Incident cases of SARS-CoV-2 infection

Participants were followed by messages, telephone calls, and medical visits from the date of the third dose (September 18th, 2021) until February 28th, 2022 to identify cases of SARS-CoV-2 infection. SARS-CoV-2 infection was confirmed by RT-PCR or a positive antigen test. All subjects with SARS-CoV-2 infection during follow-up until February 28th, 2022, were interviewed via telephone using a structured questionnaire on SARS-CoV-2 infection. Information related to the complications of acute COVID-19 infection, such as hospitalization, death, and reinfection, was recorded. Thereafter, ARD patients and non-ARD controls were interviewed by telephone approximately five months later for post-COVID-19 syndrome assessment using a structured questionnaire.

### Post-acute COVID-19

A specific structured questionnaire was designed and applied based on the World Health Organization’s post-COVID-19 condition assessment guide [11] and adapted based on relevant questionnaires published in the literature [5]. Thirty-four symptoms were systematically evaluated, including fatigue, myalgia, arthralgia, headache, dizziness, fever, cough, dyspnea, wheezing, chest pain, palpitation, increased blood pressure, thrombosis, tingling, red spots on the skin, hair loss, changes in menstrual cycle, loss of smell, loss of taste, loss of appetite, constipation, diarrhea, abdominal pain, weight loss, pollakiuria, dysuria, memory loss, mood change, insomnia, oversleeping, nightmares, concentration deficit, anxiety, and depression. Duration of each symptom was recorded in days. New or aggravated pre-existing symptoms and the resolution time (in days) for each symptom were recorded. Post-acute COVID-19 was considered when a new symptom occurred or aggravated pre-existing symptom persisted for at least 4 weeks (28 days) after the onset of acute SARS-CoV-2 infection symptoms [2, 12]. We also consider for analysis a subgroup of > 12 weeks post-acute COVID-19 when a new symptom occurred or an aggravated pre-existing symptom persisted for at least 90 days after the onset of acute SARS-CoV-2 infection symptoms [2].

### Statistical analysis

Continuous variables were presented as mean (standard deviation), and median (minimum value and maximum value) and were compared by the Student’s *t*-test or the Mann Whitney U test, as applicable. Categorical variables were presented as numbers (percentages) and were compared using Fisher’s exact test. Statistical significance was set at *p* < 0.05.

## Results

### Study participants

From the total of 1048 ARD patients and 428 non-ARD controls initially recruited for the study, 804 ARD patients and 213 non-ARD controls completed the interview (Additional file 1: Fig. S1). Of the 136 patients and 37 controls with reported COVID-19, 28 ARD patients and five non-ARD controls were excluded from the present study due to the following reasons: vaccination with other vaccine platforms (14 ARD and 3 non-ARD controls), SARS-CoV-2 infection not confirmed by RT-PCR or antigen test (13 ARD and 2 non-ARD controls), and death during the study (1 ARD). The final group for the post-acute COVID-19 analysis included 108 ARD patients and 32 non-ARD controls (Additional file 1: Fig. S1).

### Five months observation period of post-acute COVID-19 in ARD patients and non-ARD controls after a third dose of CoronaVac vaccine in SARS-CoV-2 infected subjects

ARD patients and non-ARD controls with post-acute COVID-19 had comparable age and sex (*p* > 0.05), including the same frequency of patients aged ≥ 60 years (27.8% vs. 25%, *p* = 0.8245). The frequency of post-acute COVID-19 was higher than 50% in both groups, with no differences between the groups (58.3% vs. 53.1%, *p* = 0.6854). The distribution of the number of symptoms was also comparable between the two studied groups (*p* > 0.05), and in both groups, most of the subjects had ≥ three symptoms. (Table 1). Regarding > 12 weeks post-acute COVID-19, a high frequency was also registered for both patients and controls without differences between groups (39.8% vs. 46.9%, *p* = 0.5419). The distribution of the number of symptoms was also similar, with a predominance of three or more manifestations (Table 1). Notably, most participants with post-acute COVID-19 developed > 12 weeks post-acute COVID-19: 68.3% (43/63) of ARD patients and 88.2% (15/17) of non-ARD controls (*p* = 0.1322).

**Table 1:** Post-acute COVID-19 in autoimmune rheumatic diseases patients and controls after a third dose of the CoronaVac vaccine in SARS-CoV-2 infected subjects

Variables	ARD patients (n = 108)	Non-ARD controls (n = 32)	*p* value
Demographics			
Current age, years	50.1 ± 13.8	46.9 ± 13.0	0.2550
Age ≥ 60 years (%)	30 (27.8)	8 (25.0)	0.8245
Female (%)	81 (75.0)	21 (65.6)	0.3655
ARD diseases*			
Rheumatoid arthritis (%)	29 (26.9)	–	–
Axial spondyloarthritis (%)	26 (24.1)	–	–
Systemic lupus erythematosus (%)	26 (24.1)	–	–
Systemic vasculitis (%)	6 (5.6)	–	–
Systemic autoimmune myopathies (%)	6 (5.6)	–	–
Primary Sjögren’s syndrome (%)	5 (4.6)	–	–
Primary antiphospholipid syndrome (%)	4 (3.7)	–	–
Systemic sclerosis (%)	6 (5.6)	–	–
Post-acute COVID-19 (≥ 4 weeks)	63 (58.3)	17 (53.1)	0.6854
Number of symptoms, n (min–max)	3 (1–24)	2 (1–15)	0.6123
1 symptom (%)	18 (28.6)	4 (23.5)	0.7686
2 symptoms (%)	11 (17.5)	6 (35.3)	0.1776
> 3 symptoms (%)	34 (54.0)	7 (41.2)	0.7886
Post-acute COVID-19 (> 12 weeks)	43 (39.8%)	15 (46.9)	0.5419
Number of symptoms, n (min–max)	3 (1–22)	2 (1–15)	0.3770
1 symptom (%)	11 (25.6)	6 (40.0)	0.3336
2 symptoms (%)	9 (20.9)	3 (20.0)	> 0.9999
> 3 symptoms (%)	23 (53.5)	6 (40.0)	0.7675

Results are expressed in number (%), mean ± standard deviation and median (minimum value-maximum value). The comparisons were performed by Fisher’s exact test for categorical variables. For the continuous variables, the *t*-test or the Mann Whitney U test were used, as applicable. (*) According to the specific criteria for each disease, as detailed in Silva et al. 2022 [13]

The frequency of post-acute COVID-19 in ARD was above 50% for all diseases analyzed, except for primary Sjögren’s syndrome (40%). Systemic sclerosis and primary antiphospholipid syndrome had the highest proportion of patients [100% (6/6) and 75% (3/4), respectively]. Considering > 12 weeks post-acute COVID-19, the highest proportion of patients was recorded in systemic autoimmune myopathies [66.7% (4/6)] and systemic sclerosis [66.7% (4/6)] (Additional file 1: Table S1).

Further analysis of risk factors for post-acute COVID-19 in ARD patients was performed by comparing 63 ARD patients with post-acute COVID-19 and 45 ARD patients without post-acute COVID-19, and no demographic data (age, sex and age ≥ 60 years), hospitalization, or reinfection were associated with post-acute COVID-19 (*p* > 0.05) (Additional file 1: Table S1). The possible contribution of different ARD to post-acute COVID-19 was also analyzed, and no association was observed for specific diseases or for the ARD as a group (*p* > 0.05). The exception were systemic sclerosis patients, with all 6 patients developing post-acute COVID-19 [9.5% (6/63) vs. 0% (0/45), *p* = 0.0398] (Additional file 1: Table S1).

### Manifestations of post-acute COVID-19 in ARD patients and non-ARD controls after a third dose of CoronaVac vaccine in SARS-CoV-2 infected subjects

The evaluation of symptoms of post-acute COVID-19 revealed that fatigue, memory loss, cough, myalgia, hair loss, dyspnea, arthralgia, mood swings and headache were the most frequent symptoms and were present in more than 10% of ARD patients, which were comparable to that of non-ARD controls (*p* > 0.05) (Table 2).

**Table 2:** Manifestations of post-acute COVID-19 in autoimmune rheumatic diseases patients and controls after third dose of the CoronaVac vaccine in SARS-CoV-2 infected subjects

Symptoms	ARD patients (n = 108)	Controls (n = 32)	*p* value
Fatigue (%)	24 (22.2)	10 (31.3)	0.2956
Memory loss (%)	19 (17.6)	6 (18.8)	0.8806
Cough (%)	18 (16.7)	4 (12.5)	0.5695
Myalgia (%)	17 (15.7)	3 (9.4)	0.3661
Hair loss (%)	16 (14.8)	5 (15.6)	0.9102
Dyspnea (%)	14 (13.0)	4 (12.5)	0.9452
Arthralgia (%)	12 (11.1)	3 (9.4)	0.7803
Mood change (%)	12 (11.1)	3 (9.4)	0.7803
Headache (%)	11 (10.2)	5 (15.6)	0.3956
Change in taste (%)	9 (8.3)	2 (6.3)	0.7005
Change in smell (%)	9 (8.3)	2 (6.3)	0.7005
Insomnia (%)	8 (7.4)	3 (9.4)	0.7164
Palpitation (%)	8 (7.4)	2 (6.3)	0.8233
Loss of concentration (%)	8 (7.4)	5 (15.6)	0.1595
Loss of appetite (%)	8 (7.4)	2 (6.3)	0.8233
Anxiety (%)	7 (6.5)	3 (9.4)	0.5767
Dizziness (%)	7 (6.5)	3 (9.4)	0.5767
Tingling (%)	6 (5.6)	2 (6.3)	0.8818
Weight loss (%)	5 (4.6)	0	0.2152
Diarrhea (%)	5 (4.6)	1 (3.1)	0.7120
Wheezing (%)	4 (3.7)	0	0.2694
Chest pain (%)	4 (3.7)	1 (3.1)	0.8769
Abdominal pain (%)	4 (3.7)	4 (12.5)	0.0597
Oversleeping (%)	3 (2.8)	1 (3.1)	0.9175
Increased blood pressure (%)	3 (2.8)	1 (3.1)	0.9175
Depression (%)	3 (2.8)	3 (9.4)	0.1056
Constipation (%)	3 (2.8)	1 (3.1)	0.9175
Urinary incontinence (%)	2 (1.9)	0	0.4381
Nightmares (%)	2 (1.9)	0	0.4381
Dysuria (%)	1 (0.9)		0.5849
Change in menstrual cycle (%)	0	0	–
Fever (%)	0	0	–
Thrombosis (%)	0	0	–
Other symptoms not listed (%)	8 (7.4)	1 (3.1)	0.6842

Results are expressed in number (%). The comparisons were performed by Fisher’s exact test

## Discussion

This was the first report to demonstrate that post-acute COVID-19 occurred in more than half of ARD patients, similar to non-ARD controls. More than 68% of the participants who reported symptoms for up to 28 days remained symptomatic for ≥ three months.

The main advantage of the present study was the inclusion of patients with different ARD diagnosed and followed in a referral hospital for autoimmune diseases defined by well-established classification criteria, while other studies used the definition of the International Code of Diseases or focused on a single rheumatic disease [7, 9]. The uniformity of the longitudinal vaccination protocol with three doses in the same period for the entire cohort was also a relevant strength of the present study, as full vaccination has been suggested to contribute to the reduction of post-acute COVID-19 [13]. The limitations of the present study were the convenience sample and the use of a non-validated instrument to assess mental health, which precludes systematic analysis of anxiety and depression. Historical groups of ARD patients and non-ARD controls before vaccination were also another limitation of this report.

The incidence of post-acute COVID-19 was high in ARD patients, but with similar frequencies to that observed in non-ARD controls, suggesting that autoimmune disease was not the determining factor for the occurrence of post-acute COVID-19. Surprisingly, in spite inflammatory conditions and immunosuppression in our ARD patients, they were not more prone to evolve post-acute COVID-19, possibly due to adequate immune regulatory response after SARS-CoV-2 vaccination [13]. The high frequency reported herein was also reported in Sjögren’s syndrome patients in a multicenter study, including subjects vaccinated with several platforms [7], and in the general population from a Brazilian study including 646 individuals mostly unvaccinated at the time of SARS-CoV-2 infection [5]. In contrast to our results, lower rate of post-acute COVID-19 were reported in the general populations of England and the United Kingdom [3, 4]. The variable definition of post-acute COVID-19 in different studies [3–5, 7], the inclusion of suspected cases of COVID-19, and the methods used to obtain the information may explain in part the discrepancies in these results [3, 5].

Regarding the number of symptoms and the duration of the post-acute COVID-19, most participants had this diagnosis lasting ≥ three months, with ≥ three symptoms in previous reports [3, 4]. We confirmed this result in the general population and extended this observation to all ARD patients.

The evaluation of demographic risk factors for post-acute COVID-19 in ARD patients did not reveal a distinct profile between ARD patients with and without this condition. Similarly, we did not observe hospitalization, including intensive care unit admission, as a risk factor for post-acute COVID-19, which remains controversial in the literature [14]. The low number of hospitalizations observed in this study may preclude a definitive conclusion regarding this parameter.

Remarkably, all manifestations of post-acute COVID-19 in the present study had similar frequencies in ARD patients and non-ARD controls. In addition, both groups experienced fatigue and memory loss as the two most common conditions. Fatigue is a challenging symptom in terms of recognition and management in ARD patients with post-acute COVID-19, as it is an overlapping feature between fibromyalgia and post-acute COVID-19 [8, 15], raising the possibility that fibromyalgia and other noninflammatory musculoskeletal pain conditions may present a higher probability of developing post-acute COVID-19. Our data do not support this hypothesis because the frequency of this syndrome and its manifestations were similar between ARD patients and non-ARD controls.

In summary, our study provides novel data demonstrating that in ARD patients, the post-acute COVID-19 has demographic characteristics and patterns of system involvement very similar to those of the general population.

## Conclusion

These data suggest that the immunological and inflammatory disorders associated with ARD do not seem to be determinants of the clinical manifestations and duration of post-acute COVID-19. This data is an encouragement for rheumatologists, but it is a huge challenge for those who follow these ARD patients considering the great overlap of manifestations between the two conditions and that most of them will evolve to longer post-acute COVID-19 condition.

## Supplementary Information

**Additional file 1: Fig. S1.** Flowchart of the inclusion of patients with autoimmune rheumatic diseases and controls with COVID-19 confirmed after the third dose of vaccination with CoronaVac. **Table S1.** Analysis of risk factors for persistent post-COVID-19 syndrome in 108 patients with ARD. Results are expressed in number. The univariate analysis was performed by Fisher’s exact test. *ARD* Autoimmune rheumatic disease; *ICU* Intensive care unit.

## Data Availability

All data relevant to the study are included in the article or uploaded as supplemental information.
