# Supplementary material for: Post-acute COVID-19 in three doses vaccinated autoimmune rheumatic diseases patients: frequency and pattern of this condition
Source: Adv Rheumatol. 2023 Jun 8;63(1):26. doi: 10.1186/s42358-023-00309-z (PMC10248954; doi:10.1186/s42358-023-00309-z)
Supplement: Supplementary file 1 — Additional file 1: Fig. S1. Flowchart of the inclusion of patients with autoimmune rheumatic diseases and controls with COVID-19 confirmed after the third dose of vaccination with CoronaVac. Table S1. Analysis of risk factors for persistent post-COVID-19 syndrome in 108 patients with ARD. Results are expressed in number. The univariate analysis was performed by Fisher’s exact test. ARD Autoimmune rheumatic disease; ICU Intensive care unit. [file 42358_2023_309_MOESM1_ESM.docx]

Control group

Patients with ARD

ARD vaccinated with

third dose of the CoronaVac

(n = 1048)

Controls vaccinated with

third dose of CoronaVac

(n = 428)

**Figure 1** – Flowchart of the inclusion of patients with autoimmune rheumatic diseases (ARD) and controls with COVID-19 confirmed after the third dose of vaccination with CoronaVac.

Participants who answered the questionnaire on SARS-CoV-2 infection and post-acute COVID-19

(n = 213)

Participants who answered the questionnaire on SARS-CoV-2 infection and post-acute COVID-19

(n = 804)

Declared COVID-19

(n=136)

Declared COVID-19

(n=37)

Exclusions (n = 15)

- 3^rd^ dose was not CoronaVac (n=14)

- COVID-19-related death (n=1)

Exclusions (n = 3)

3^rd^ dose was not CoronaVac (n=3)

COVID-19 declared

(n = 2)

COVID-19 confirmed

(n = 32)

COVID-19 confirmed

(n = 108)

COVID-19 declared

(n = 13)

Controls analyzed for post-acute COVID-19

N=32

ARD patients analyzed for post-acute COVID-19

N=108

**Supplementary_Table S1 – Analysis of risk factors for post-acute COVID-19 in 108 patients with ARD**

| **Variables** | | **Post-acute COVID-19** | |  |
| --- | --- | --- | --- | --- |
|  | |  |  |  |
|  | **Present (n=63)** | | **Absent (n=45)** | **P** |
| Male (%) | 16 (25.4) | | 11 (24.4) | >0.9999 |
| Age > 60 years (%) | 20 (31.7) | | 10 (22.2) | 0.3837 |
| Hospitalization (%) | 1 (1.6) | | 1 (2.2) | >0.9999 |
| ICU hospitalization (%) | 1 (1.6) | | 0 | >0.9999 |
| Reinfection (%) | 14 (22.2) | | 6 (13.3) | 0.3174 |
| **ARD diseases** |  | |  |  |
| **Inflammatory chronic arthritis** | 31 (49.2) | | 24 (53.3) | 0.7002 |
| Rheumatoid arthritis (%) | 16/29 (25.4) | | 13/29 (28.9) | 0.8260 |
| Axial spondiloartritis (%) (n=26) | 15/26 (23.8) | | 11/26 (24.4) | >0.9999 |
| **Autoimmune diseases** | 32 (50.8) | | 21 (46.7) | 0.7002 |
| Systemic lupus erythematosus (%) | 14/26 (22.2) | | 12/26 (26.7) | 0.6514 |
| Systemic vasculitis (%) | 3/6 (4.8) | | 3/6 (6.6) | 0.6918 |
| Idiopathic inflammatory myopathies (%) | 4/6 (6.3) | | 2/6 (4.4) | >0.9999 |
| Primary Sjögren’s syndrome (%) | 2/5 (3.2) | | 3/5 (6.7) | 0.6473 |
| Primary antiphospholipid syndrome (%) | 3/4 (4.8) | | 1/4 (2.2) | 0.6392 |
| Systemic sclerosis (%) | 6/6 (9.5) | | 0 | **0.0398** |

Results are expressed in number (%). The univariate analysis was performed by Fisher’s exact test. ARD: Autoimmune rheumatic disease; ICU: Intensive Care Unit
